# The Indications for Infusion

**Published:** 1910-07-02

**Authors:** 


### Surgery.

### THE INDICATIONS FOR INFUSION.

Heart failure in both shock and collapse results from the failure of the coronary circulation, which depends upon the difference of pressure at the base of the aorta and in the great veins. The cause of the fall of blood-pressure in shock is, however, different from what it is in collapse, and this is the essential difference between the two conditions. Shock is a general vaso-dilatation due to a temporary paresis of the vaso-motor centre caused by excessive stimulation of some sensory nerve, or by some poison acting on the centre. Hence the total volume of the whole circulatory system is greatly increased, and the blood-pressure falls. As there is a larger number of vessels in the splanchnic area than in any other part of the body, the increase of volume is most marked in this area. In shock, therefore, no fluid is lost from the circulatory system, but it is banked up in the splanchnic area instead of circulating throughout the body. In collapse, on the other hand, there is a loss of fluid from the body, or into some part of the body from which it cannot be regained into the circulatory system. Normally the tissues will give up fluid to the circulation as quickly as it is taken out from some other part, and will continue to do so until there is no more fluid in the tissues to be taken up. When, however, a tissue is damaged it rapidly takes up fluid from the blood, and cannot give it back when the latter becomes lessened in quantity.

### Conditions under which Loss of Fluids Takes Place.

Hence in collapse the fall of blood-pressure is due to fluid actually lost from the circulation, and may occur under three conditions:—

(*a*) All the constituents of the blood are lost from the body in cases of severe hæmorrhage.

(*b*) The fluid parts only of the blood are lost from the body, as in cases of diarrhœa and vomiting.

(*c*) The fluid parts are lost from the circulation, not from the body, but into some damaged tissue from which they cannot be regained, as in cases of general peritonitis or extensive burns.

In both shock and collapse the addition of fluid to the circulation will be of benefit. In shock the result of this will be to raise the blood-pressure by filling up the increase of volume of the circulatory system. But as the fluid tends to collect in the veins, the good result of infusions in this condition is temporary, and their scope is limited because the added fluid after a time goes straight into the veins without raising the blood-pressure. In collapse the result is more permanent, for the fluid replaces that which is lost and brings back the circulation to normal by filling up the arterio-venous system. Here the important point to remember is that the fluid must be put in early, for the fall of blood-pressure in this condition does not occur until all the available fluid in the tissues has been given up to the circulation. Hence the fall in blood-pressure is sudden and dangerous, and treatment should aim at preventing this rather than at remedying it when it has happened. In other words, it is important to give fluid somehow to a patient who is in a condition likely to lead to collapse, without waiting for symptoms of the collapse to show themselves.

### Infusion.

When fluid cannot be taken by the mouth its administration is known as infusion, which may be into the rectum, into the cellular tissues, or directly into the veins. It may also be given continuously or interruptedly in repeated small quantities. The fluid to be used must be iso-tonic with the blood; if it be less than this, fluid is attracted from the blood-stream to the tissues of higher osmotic pressure, and the very opposite results to what is intended. Sodium chloride, 1½ drachm to a pint of tap-water, is approximately iso-tonic with the blood; the advantage of using tap-water is that it contains some calcium salt, which is a valuable stimulant to the heart muscle.

A more continuous steady improvement is likely to occur in the condition of the patient if a slow continuous infusion be given at a low pressure than if a smaller amount be rapidly put in from time to time under a higher pressure. In children, in very restless patients, or where a continual watch cannot be kept, this latter method is to be preferred.

### Rectal Infusion.

The technique of infusion into the rectum varies according as to whether it is to be interrupted or continuous. In the former case a pint of saline is given at a time and repeated every hour or every two hours. A rubber tube is introduced for a distance of 6 to 10 inches into the bowel and the solution poured into a funnel held about 6 inches to a foot above the anus; about a quarter of an hour is taken up in giving the infusion. If it be very urgent to introduce fluid at once, as after sudden profuse hæmorrhage, a couple of pints may be put rapidly in with an ordinary Higginson's syringe, and it will be absorbed with remarkable rapidity. This method, however, cannot be used when repeated infusions are necessary, as the colon soon becomes intolerant of large quantities rapidly injected, but must be used only where a single rectal infusion is employed.

### Continuous Infusion.

When a continuous infusion into the rectum is used the ordinary vaginal nozzle that is supplied with an enema syringe is connected by some 2 yards of rubber tubing with a douche-can containing the solution at 120° F. This can is stood in a basin of hot water at such a height above the nozzle that a drop falls in about every two or three seconds; the nozzle is then inserted just within the anus. It will generally be found that the douche-can must be about 4 inches above the anus in order that it may run at the correct rate. In this way fluid is passed into the colon at the rate of half a pint per hour or less, and the gut will absorb it at this rate for three or four days on end. It is not necessary for the patients to be lying down, as they will retain the fluid at this rate even in the sitting position. This is a most valuable method of giving fluid over an extended period of time, and has the advantage of needing no very special apparatus or the sterilisation of the saline solution. It is, however, useless in the case of a restless patient, or of one who is intolerant of the nozzle in the rectum. Further, all the methods of infusion per rectum fail where the lower gut is distended with fæces, or where some hard scybalous masses are lying low down in the canal within reach of the finger.

### The Intracellular Method.

When fluid is infused into the cellular tissues the best place to put it is into loose cellular tissue of the armpit. A hollow needle is driven into the axilla till its point comes to lie just under the skin. This is best done by firmly gripping the anterior axillary fold between finger and thumb and driving the needle through skin and pectorals till the point is felt beneath the skin on the posterior side; when the point of the needle is actually felt it is known that it has not injured any important structure and that it now lies in a potential space capable of considerable distension. The pectoral muscles act as splints to keep the needle upright. The needle is now attached to some apparatus of which the simplest form is a piece of rubber connected with a bent glass tube which syphons the fluid out of a glass receptacle. This is held at varying distances above the needle according to the rate at which it is required to introduce the fluid. For a continuous infusion the fluid should be only just raised above the needle; for an interrupted one the fluid is raised higher and the tissues filled with fluid; the needle is then withdrawn and the fluid allowed time to absorb. The apparatus of Lane and Barnard are both excellent. Lane's rubber bag has the advantage that the fluid can be sterilised in it. Barnard's apparatus connects with two finer needles which can be put one into each thigh. In small children suffering from severe diarrhœa only a very small amount of fluid can be introduced at once; it can then be put into the cellular tissues of the abdomen or chest by means of an exploring syringe, an ounce or less being given at a time, and repeated at frequent intervals.

### Intravenous Infusion.

The third method of introducing fluid is by putting it straight into the circulation by means of a vein. In doing this the vein may be stabbed with a hollow needle, but it is better to dissect out the vessel and tie in a blunt-ended cannula connected with a receptacle containing the fluid. Generally one of the veins lying in front of the bend of the elbow is chosen. The operation is done as follows: An incision 1 inch long is made parallel with the vein which is cut down upon and dissected out for about ½ inch. A double ligature is then passed round it, and the distal one is firmly tied; one hitch is loosely tied around the proximal. The vessel wall is now half severed with a pair of scissors, and into the opening so made the cannula is inserted in an upward direction and the loose hitch of the ligature tightened on it to retain it in place and to prevent the escape of fluid. The fluid is now slowly run in, and anything up to 3 pints may be given at a rate not quicker than a pint in a quarter of an hour. Great care must be taken not to introduce any air. When all the fluid needed has been put in, the cannula is removed and the ligature tied around the vein, which is divided between the two ligatures. The wound is then bound over with a pad, or a suture is put in. In both these last methods great care must be taken to prevent sepsis; the salt should be added to the water and the solution well boiled for ten minutes and allowed to cool, or the salt can be boiled in a stronger solution and sterile water added to cool it to 120° F. In the event of cold sterilised water not being available the water from the hot tap of a circulating system in a house may be used, as this is approximately sterile. If an urgent case will not admit of the inevitable delay in a private house of sterilising and cooling, a pint of saline may be given per rectum and one of the latter methods employed when the solution is ready. The skin area may be cleaned with soap and water and methylated spirit, or painted with tincture of iodine.

Infusion in one way or another will be found of the greatest value in cases of burns or injuries in which blood has been lost, and in all cases of appendicitis or peritonitis from other causes even when these are not subjected to operation. After a laparotomy this means of treatment is made the rule by some surgeons with very good results.

